# Leaf trait plasticity reveals interactive effects of temporally disjunct grazing and warming on plant communities

**DOI:** 10.1007/s00442-024-05540-z

**Published:** 2024-04-04

**Authors:** Argo Ronk, Bazartseren Boldgiv, Brenda B. Casper, Pierre Liancourt

**Affiliations:** 1 Department of Biology, University of Pennsylvania, Philadelphia, USA; 2 Department of Biology, National University of Mongolia, Ulaanbaatar, 14201 Mongolia; 3 Department of Botany, State Museum of Natural History Stuttgart, Stuttgart, Germany; 4 Department of Evolution and Ecology, University of Tübingen, Tübingen, Germany

**Keywords:** Climate change, Community-weighted mean, Functional diversity, Grazing, Intraspecific trait variation

## Abstract

Changes in climate and grazing intensity influence plant-community compositions and their functional structure. Yet, little is known about their possible interactive effects when climate change mainly has consequences during the growing season and grazing occurs off growing season (dormant season grazing). We examined the contribution of trait plasticity to the immediate responses in the functional structure of plant community due to the interplay between these two temporally disjunct drivers. We conducted a field experiment in the northern Mongolian steppe, where climate was manipulated by open-top chambers (OTCs) for two growing seasons, increasing temperature and decreasing soil moisture (i.e., increased aridity), and grazing was excluded for one dormant season between these two growing seasons. We calculated the community-weighted mean (CWM) and the functional diversity (FD) of six leaf traits. Based on a variance partitioning approach, we evaluated how much of the responses in CWM and FD to OTCs and dormant season grazing occur through plasticity. The interactive effect of OTCs and the dormant season grazing were detected only after considering the role of trait plasticity. Overall, OTCs influenced the responses in CWM more than in FD, but the effects of OTCs were much less pronounced where dormant season grazing occurred. Thus, warming (together with decreased soil moisture) and the elimination of dormant season grazing could interact to impact the functional trait structure of plant communities through trait plasticity. Climate change effects should be considered in the context of altered land use, even if temporally disjunct.

**Supplementary Information:**

The online version contains supplementary material available at 10.1007/s00442-024-05540-z.

## Introduction

Understanding how biological communities will respond to global change is a major challenge in ecology (Sala et al. 2000; Suding et al. 2008). A growing number of studies has demonstrated that community responses are difficult to predict without considering explicitly the complex and interactive effects of climate and land use drivers (Grime et al. 2000; Christensen et al. 2004; Klein et al. 2004, 2007; Pyke and Marty 2005; Diaz et al. 2007). In drylands, continued aridification and changes in grazing regime are expected to produce significant consequences for ecosystem functions (Maestre et al. 2016; Vandandorj et al. 2017; Gaitán et al. 2018; Nandintsetseg et al. 2021; Maestre et al. 2022), by altering species occurrence and abundance, ultimately changing the functional structure of plant communities, i.e., the distribution of plant trait values (Suding et al. 2008; Valencia et al. 2015; Funk et al. 2017; Gross et al. 2017; Le Bagousse-Pinguet et al. 2017). While most of this knowledge comes from studies where the main consequences of climate change and grazing pressure occurred concurrently during the growing season, grazing during fall or winter is also a common rangeland management practice influencing plant communities (e.g., Caballero et al. 2022). Little is known about possible interactive effects of grazing and climate change when these global change drivers are temporally disjunct, that is, when climate change effects on plants are most expressed during the growing season and grazing occurs when plants are dormant.

The functional structure of plant communities can change through interspecific and intraspecific trait variation (de Bello et al. 2011; Lepš et al. 2011). Interspecific responses reflect changes in the representation of species with different trait values, while intraspecific responses reflect changes in trait phenotypes due to replacement of genotypes and/or phenotypic plasticity (Nicotra et al. 2010; Bolnick et al. 2011; Siefert et al. 2015). Earlier trait-based studies have typically ignored intraspecific variation, assuming, it is much lower than interspecific variation and that species-rankings based on functional traits are unaffected by spatio-temporal variation (Garnier et al. 2001) or well correlated between different management regimes (Mudrák et al. 2019), (but see Albert et al. 2010; Jung et al. 2014; Siefert et al. 2015). Yet, countless studies have demonstrated that intraspecific trait variation can be considerable, with significant ecological implications (Nicotra et al. 2010; Bolnick et al. 2011), and can be a key indicator of subsequent changes in species abundance (Liancourt et al. 2015) and community dynamics (Griffiths et al. 2018). Untangling the relative contributions of inter- and intraspecific trait variation, and particularly the role of plasticity, is essential in understanding plant-community responses to environmental change (Nicotra et al. 2010; Violle et al. 2012; Wang et al. 2022).

Intraspecific and interspecific variation could covary in two ways, as a negative correlation, possibly indicating that trait plasticity buffers the effect of environmental change on plant-community composition, or as a positive correlation, reinforcing interspecific responses (de Bello et al. 2011; Lepš et al. 2011). Covariation may depend on the particular trait (e.g., Roos et al. 2019) or the particular attribute of the functional community structure considered, such as the mean trait value or its variance/diversity. Evidence so far mostly suggests a positive covariation between intraspecific and interspecific trait variation, as revealed by the community-weighted mean (Lepš et al. 2011; Siefert et al. 2015), although see de Bello et al. (2011). The relative roles, or magnitude, of intraspecific vs. interspecific variation on functional diversity (FD) and the direction of their covariation are still largely unknown. For these reasons, pooling functional trait variation within a species could bias our estimation of the rate and magnitude of community and ecosystem responses (see Jung et al. 2014).

Grassland ecosystems are projected to be severely impacted by both global environmental change and altered land use (Sala et al. 2000). Providing forage for vertebrate grazers is a primary land use in grassland ecosystems, but grazing regimes are changing with human cultural practices (Kahmen and Poschlod 2008). Grazing can greatly influence standing biomass, litter accumulation, community composition, and carbon and nutrient cycles (Facelli and Pickett 1991; Peco et al. 2012; Spence et al. 2014; Kohli et al. 2021) and be of key importance in plant-community responses to climate change (Pyke and Marty 2005; Klein et al. 2007; Post and Pedersen 2008; Maestre et al. 2022). Some evidence suggests that aridity and grazing can select for similar traits and that aridity can promote resistance to grazing (Milchunas et al. 1988; Adler et al. 2004; Quiroga et al. 2010; Koerner and Collins 2014). Nevertheless, most studies have investigated combined effects of grazing and climate change occurring concurrently during the growing season. Much less attention has been paid to systems with dormant season grazing, decoupled from the growing season, when animals rely on plant litter produced by the preceding seasonal growth.

We combined climate manipulation achieved through passive open-top chambers—OTCs, which increase air temperature and decrease soil moisture in our system (i.e., increased aridity, see Liancourt et al. 2012a, b; Ronk et al. 2020), with the cessation of the traditional non-growing season grazing in the Mongolian steppe. This steppe is part of the world’s largest, yet understudied, expanse of grassland in a region historically known for pastoralism (Christensen et al. 2004; Spence et al. 2014). More than three quarters of Mongolia’s land area is used for grazing livestock, making herding the main source of livelihood in rural areas (Batima et al. 2013). Yet, traditional nomadic pastoralism is shifting to more sedentary pastoralism and is threatened by urbanization, which, in turn, results in increased grazing intensity in some areas and decreased grazing, or even grazing abandonment, in others (Morris and Bruun 2005).

Hence, we examined the effect of OTC and grazing cessation on the community-weighted mean (CWM) and the FD, which are two distinct aspects of the community functional structure that may respond to the treatments (e.g., Le Bagousse-Pinguet et al. 2017). We calculated CWM of some common traits of the most abundant species (see Lepš and de Bello 2023 for recent review), and FD, the community-wide variation in a trait (e.g., Valencia et al. 2015 and references therein). We assessed for both CWM and FD the contributions made by changes in interspecific trait values due to species turnover or by changes in intraspecific trait values (de Bello et al. 2011; Lepš et al. 2011).

We measured six commonly assessed functional leaf traits: Leaf area (LA), leaf length (LL), specific leaf area (SLA), leaf dry matter content (LDMC), leaf nitrogen content (LNC), and leaf carbon content (LCC) (Appendix S1). Since productivity in this mountain steppe is co-limited by temperature and water availability (Liancourt et al. 2012a), we expected the warmer and drier conditions in the OTC (see Ronk et al. 2020) to favor conservative strategies like low LNC, low SLA, and high LDMC and smaller LA, with reduced transpiration surfaces (Ordoñez et al. 2009; Pierce et al. 2013; Le Bagousse-Pinguet et al. 2017; Kramp et al. 2022). Given that grasses have been shown to grow taller with higher SLA and higher LNC under higher temperature (Jardine et al. 2020; Sandel et al. 2021) and our system is dominated by the graminoids, we alternatively expected non-conservative responses of the graminoids in the OTCs to influence the CWM of the assessed traits accordingly. A decrease in FD in the OTC could be expected if warming and decreased soil moisture favor particular/optimal trait values and disfavor other trait values. An increase in FD in the OTC would occur if species with different trait values are equally favored or if plastic traits respond in different directions in different species (see also Le Bagousse-Pinguet et al. 2017; Griffin-Nolan and Sandel 2023). We predicted grazing to affect the measured leaf traits as well, because some species could be selectively foraged (species with high LNC and low LDMC) or because some leaf traits respond to shading (e.g., higher LA, higher LL, higher SLA) from standing plant litter in the un-grazed treatment (Spence et al. 2014).

## Materials and methods

### Study site

This study was conducted in 2009 and 2010 on a south-facing slope within mountain steppe in the Dalbay River valley (51°01.405′N, 100°45.600′E). The coldest average monthly temperature is −21 °C (Jan.) and the warmest 12 °C (July). On-site total annual precipitation (Jan. to Dec.) was 270 mm in 2009 and 246 mm in 2010, and most of the precipitation was recorded during the short growing season (early June to the end of August) in each year (see Liancourt et al. 2012a, b). The valley is primarily grazed during the non-growing season (end of August–end of May) by yaks, horses, sheep, and goats. The vegetation is a mixture of sedges (e.g., *Carex* spp.), grasses (e.g., *Festuca lenensis, Koeleria macrantha*), and forbs (e.g., *Aster alpinus, Potentilla acaulis*). Due to difficulties with identifications, cover data were combined for *Carex* spp., representing *C. pediformis*, *C. dichroa,* and *C. duriuscula*, and for *Allium* spp. representing *A. bidentatum* or *A. prostratum*. The only woody species are dwarf shrubs, *Thymus gobicus* and *Artemisia frigida*.

### Experimental design

At ~1670 m elevation, on the lower slope of the valley (flat to gentle incline), we applied the factorial combination of the OTC during the growing season and dormant season grazing: OTC with grazing, OTC without grazing, grazing without OTC, and neither, with one replicate of each treatment in each of eight blocks. A block consisted of a 9 × 9 m area fenced year-round and an adjacent 3 × 9 m area, where fencing was removed from three sides in mid-August 2009, to allow local herd animals access, and re-installed in early June 2010 before the next growing season. One OTC was in place in each area from early June to mid-August 2009 and 2010. Another plot in each area, with the same dimensional footprint as the area covered by the OTC, was permanently marked. This arrangement provided the factorial combinations of grazing and climate manipulation. The blocks were scattered roughly in a row over 321 m.

OTCs were constructed with Sun-Lite® HP fiberglass glazing mounted on a clear Lexan frame (Marion et al. 1997). They were hexagonal with slanted sides, 1.0 m wide at the top, 1.5 m at the bottom, and 40 cm tall. OTCs elevated air temperatures on average by 1.5 °C in the day and depressed it by −0.2 °C at night (Liancourt et al. 2012b). By intercepting rain, OTCs also elevated soil temperature and decreased soil moisture (by ~30%; Liancourt et al. 2012a; Ronk et al. 2020).

### Plant community censuses and leaf functional traits

Percent cover by species was determined in mid-July 2010, using a 50 cm × 100 cm quadrat, string-gridded into 10 cm × 10 cm cells, with the short side of the rectangle parallel with the northern side of the plot (see also Ronk et al. 2020). Percentage cover per species was estimated in each of the 50 cells to the nearest 10% and averaged over the entire quadrat (i.e., all 50 cells) to estimate species percent cover per plot.

Leaf functional traits were measured in July 2010 for the most common species, which altogether represented more than 96% of the vegetation cover across all plots (Appendix S2). The measured traits were LA, SLA, LL, LDMC, and LNC and LCC. A total of three to ten fully developed leaves were collected from one to three adult individuals of each species in each plot, depending on species abundance and leaf size, and combined for trait measurements to yield a single value for each trait per species per plot. Measurements followed standard protocols (Perez-Harguindeguy et al. 2013) except for LDMC, where fresh mass was measured using a partial dehydration method in the field after leaves were kept overnight in a moist paper towel at ambient temperature (Vaieretti et al. 2007). Dry mass was measured on oven-dried leaves (48 h, 80 °C) in the laboratory. Traits measured on forbs included petioles. Protocol details can be found in Liancourt et al. (2015).

### Inter- vs. intraspecific trait variation and covariation

We calculated the CWM, and FD for every plot, using log-transformed trait values in each case. FD was calculated using the Rao index (Rao 1982; Botta-Dukát 2005). To estimate trait responses attributable to interspecific trait variability (Inter), intraspecific trait variability (Intra), and their combined effect (Intra + Inter), we followed the method of Lepš et al. (2011) for CWM and de Bello et al. (2011) for FD. First, we calculated CWM and FD for each plot based on species’ “fixed” trait values (sensu de Bello et al. 2011; Lepš et al. 2011), which for a given species is the mean value computed from values in all experimental plots in which that species occurred. Using fixed trait values completely neglects the extent of intraspecific trait variability between treatments; therefore, variation in CWM and FD across plots can only result from differences in species composition. Second, we calculated CWM and FD values based on species “specific” trait values (sensu de Bello et al. 2011; Lepš et al. 2011) using the mean trait value of a species for a specific treatment. We then calculated intraspecific trait variability as the difference between “specific” trait values and “fixed” trait values (sensu de Bello et al. 2011; Lepš et al. 2011). Finally, by decomposing total variance, we examined the covariation, either positive or negative, between interspecific and intraspecific trait variation (for details see, de Bello et al. 2011; Lepš et al. 2011).

To evaluate the effect of the OTC, grazing, and their interaction on inter- and intraspecific trait variation and their combined effect (Intra + Inter), we conducted distinct two-way ANOVAs for each of the three community level parameters for each leaf functional trait. We performed all statistical tests using R ver. 4.3.2 (R Core Team 2017) and using R scripts provided by Lepš et al. (2011) and dbFD function in the ‘FD’ package for calculation of Rao’s index (Laliberté and Legendre 2010).

## Results

**Fig. 1:**
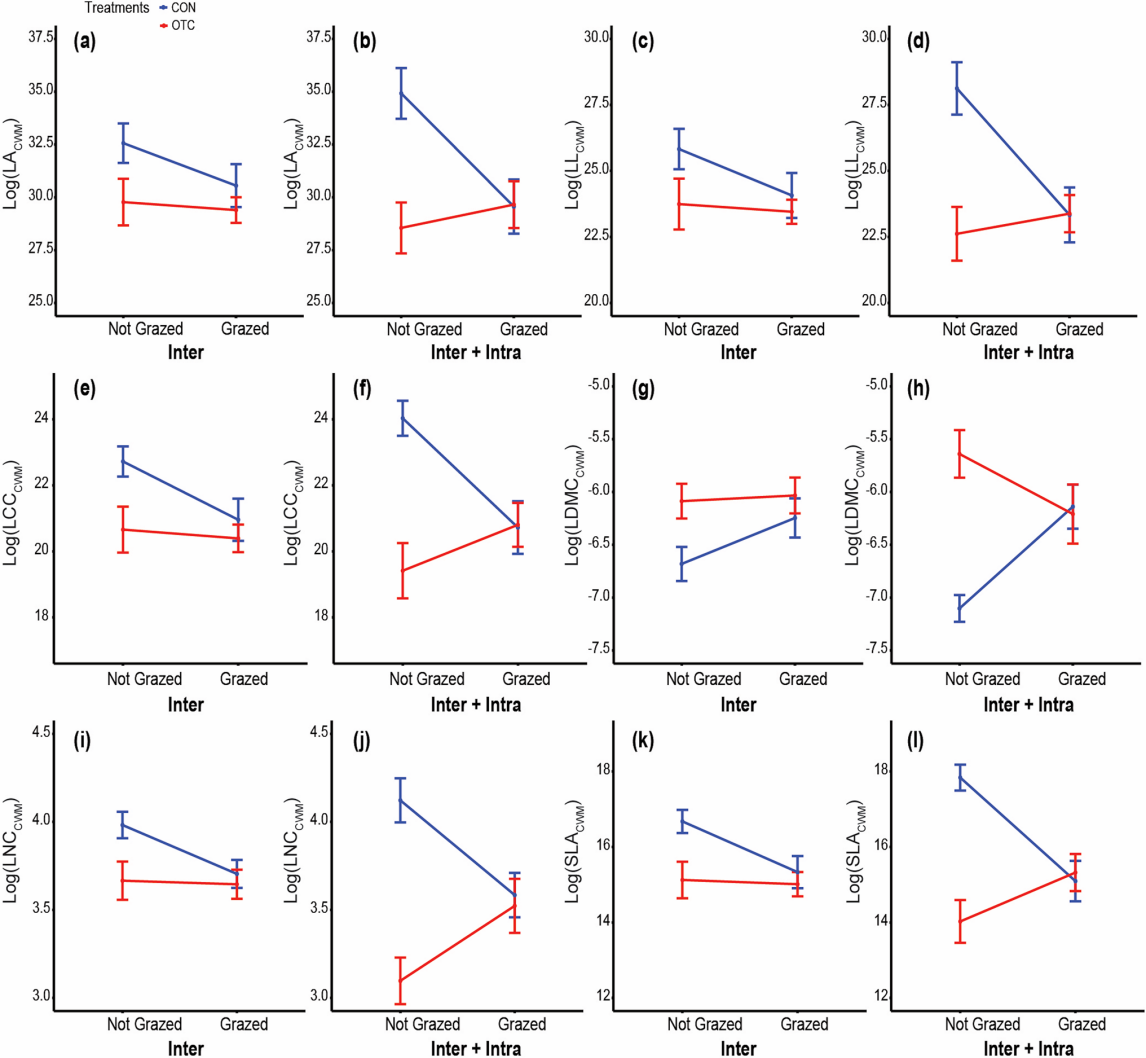
Community-weighted means (log-transformed) for six leaf functional traits: **a** and **b** Leaf area, **c** and **d** Leaf length, **e** and **f** Leaf carbon content, **g** and **h** Leaf dry matter content, **i** and **j** Leaf nitrogen content, **k** and **l** Specific leaf area, in the four combinations of OTC and grazing. The interspecific variability (Inter) averages are based on species trait values that are averaged over all treatments, so variation between treatments in (Inter) CWM reflects variation in species representation. Both differences in species abundances and treatment induced changes in intraspecific variability (i.e., plasticity) contribute to (Inter + Intra) CWM for a given combination of OTC and grazing. Error bars denote standard errors

Overall, OTC shifted communities toward smaller leaves (lower LA_CWM_, marginally significant lower LL_CWM_, Table 1, Fig. 1), and a more conservative syndrome with respect to other traits (i.e., higher LDMC_CWM_, lower LNC_CWM_, lower SLA_CWM_, Table 1, Fig. 1). The OTC also decreased LCC_CWM_. These shifts were the consequence of the combined effect of interspecific and intraspecific sources of variation, with intraspecific variation amplifying the effect of OTC expressed through interspecific variation as they were positively correlated (Appendix S3). Dormant season grazing, as a main effect, was only significant for LL_CWM_ (shorter leaves), and primarily through intraspecific variation (Table 1). However, and importantly, with interspecific and intraspecific sources of variation combined, there was a strong-interactive effect of OTC and grazing (OTC × grazing interaction; Table 1, Fig. 1) where traits showed a pronounced response to the OTC only in the absence of dormant season grazing. This interaction was undetected when considering the interspecific level (i.e., change in species composition) as the only source of variation influencing CWM (Table 1). Thus, intraspecific sources of variation in leaf traits amplified the mitigating effect of grazing on the community response to OTC, i.e., amplified the response to grazing cessation without OTC (Fig. 1).

**Table 1:** Results of two-way ANOVAs for the community-weighted mean (CWM) of leaf functional traits

		Inter			Inter + Intra			Intra		
		SS	*F*	*P*	SS	*F*	*P*	SS	*F*	*P*
LA_CWM_	OTC	31.17	4.47	**0.043**	78.71	6.83	**0.014**	10.82	3.21	0.083
	Grazing	11.40	1.63	0.212	36.21	3.14	0.087	6.97	2.07	0.161
	OTC × Grazing	5.34	0.77	0.389	83.54	7.25	**0.012**	46.65	13.86	**<0.001**
LL_CWM_	OTC	14.58	2.98	0.096	59.53	8.27	**0.008**	15.19	9.12	**0.005**
	Grazing	8.36	1.71	0.202	32.46	4.51	**0.043**	7.88	4.73	**0.038**
	OTC × Grazing	4.29	0.88	0.36	61.76	8.58	**0.007**	33.49	20.12	**<0.001**
LCC_CWM_	OTC	13.82	5.39	**0.027**	41.19	10.01	**0.004**	7.29	6.09	**0.020**
	Grazing	8.26	3.22	0.083	7.38	1.79	0.191	0.02	0.02	0.887
	OTC × Grazing	4.50	1.76	0.196	44.18	10.74	**0.003**	20.48	17.13	**<0.001**
LDMC_CWM_	OTC	1.30	5.60	**0.025**	3.88	10.26	**0.003**	0.69	3.91	0.057
	Grazing	0.48	2.06	0.163	0.31	0.82	0.372	0.02	0.10	0.754
	OTC × Grazing	0.29	1.25	0.273	4.70	12.42	**0.001**	2.65	15.09	**<0.001**
LNC_CWM_	OTC	0.28	4.61	**0.040**	2.36	16.20	**<0.001**	1.01	15.78	**<0.001**
	Grazing	0.18	2.91	0.099	0.03	0.17	0.681	0.07	1.08	0.308
	OTC × Grazing	0.13	2.17	0.152	1.86	12.75	**0.001**	1.0	15.57	**<0.001**
SLA_CWM_	OTC	7.0	5.70	**0.024**	25.72	13.32	**0.001**	5.88	9.11	**0.005**
	Grazing	4.25	3.46	0.073	4.19	2.17	0.152	**<**0.001	**<**0.001	0.987
	OTC × Grazing	3.04	2.47	0.127	32.65	16.91	**<0.001**	15.77	24.42	**<0.001**

Effects through interspecific (Inter), intraspecific (Intra), and Inter + Intra variability analyzed separately. SS denotes Sum of Squares. Significant *p*-values (*p*** < **0.05) indicated in bold

How FD responded to the OTC and grazing treatments differed among leaf traits, both in magnitude and direction of responses. Regardless, responses were expressed exclusively through intraspecific variation. There was no effect of OTC, grazing, or their interaction on FD expressed solely through interspecific variation, for any trait (Table 2). Through intraspecific variation, the overall effect of OTC significantly decreased SLA_FD_ and LNC_FD_, and increased LDMC_FD_. The overall effect of grazing cessation decreased LNC_FD_. We also found several traits showing significant OTC × grazing interactions: OTC and grazing cessation decreased LA_FD_ and LL_FD_ compared to the control conditions with grazing, and LCC_FD_ increased with grazing cessation only without OTC (Table 2, Appendix S4). Yet, the response of the intraspecific component of FD to the treatments and the treatment interaction accounted for only a little of the variation in FD observed (Appendix S5). In other words, the effects of our treatments on the intraspecific component of FD for LL, LA, LDMC and SLA, while significant, were, in fact, negligible.

**Table 2:** Results of two-way ANOVAs for the functional diversity (FD) of leaf functional traits

		Inter			Inter + Intra			Intra		
		SS	*F*	*P*	SS	*F*	*P*	SS	*F*	*P*
LA_FD_	OTC	0.004	0.06	0.813	0.010	0.14	0.709	0.028	20.25	**<0.001**
	Grazing	0.027	0.36	0.554	0.008	0.12	0.737	0.005	3.93	0.057
	OTC × Grazing	0.008	0.11	0.744	<0.001	<0.001	0.985	0.009	6.63	**0.016**
LL_FD_	OTC	0.003	0.02	0.883	0.023	0.15	0.702	0.043	13.05	**0.001**
	Grazing	0.013	0.09	0.765	0.430	2.74	0.109	0.294	88.29	**<0.001**
	OTC × Grazing	0.019	0.14	0.716	0.106	0.68	0.417	0.035	10.60	**0.003**
LCC_FD_	OTC	0.004	0.15	0.702	0.016	0.29	0.596	0.036	2.16	0.153
	Grazing	0.001	0.05	0.833	0.633	11.46	**0.002**	0.691	41.11	**<0.001**
	OTC × Grazing	0.001	0.03	0.867	0.620	11.21	**0.002**	0.576	34.25	**<0.001**
LDMC_FD_	OTC	0.002	0.01	0.932	0.193	0.86	0.361	0.155	17.52	**<0.001**
	Grazing	0.037	0.13	0.718	0.064	0.29	0.596	0.003	0.42	0.523
	OTC × Grazing	0.073	0.26	0.613	0.018	0.08	0.778	0.018	2.05	0.162
LNC_FD_	OTC	0.211	1.79	0.191	0.001	0.02	0.881	0.178	5.62	**0.025**
	Grazing	0.002	0.01	0.904	0.268	4.47	**0.044**	0.313	9.89	**0.004**
	OTC × Grazing	0.034	0.29	0.595	0.004	0.061	0.805	0.060	1.90	0.179
SLA_FD_	OTC	<0.001	<0.001	0.978	0.173	3.019	0.093	0.178	8.75	**0.006**
	Grazing	0.026	0.74	0.398	<0.001	<0.001	0.981	0.027	1.35	0.256
	OTC × Grazing	0.020	0.58	0.452	0.030	0.53	0.474	0.001	0.05	0.825

Effects through interspecific (Inter), intraspecific (Intra), and Inter + Intra variability analyzed separately. SS denotes Sum of Squares. Significant *p*-values (*p*** < **0.05) indicated in bold

**Fig. 2:**
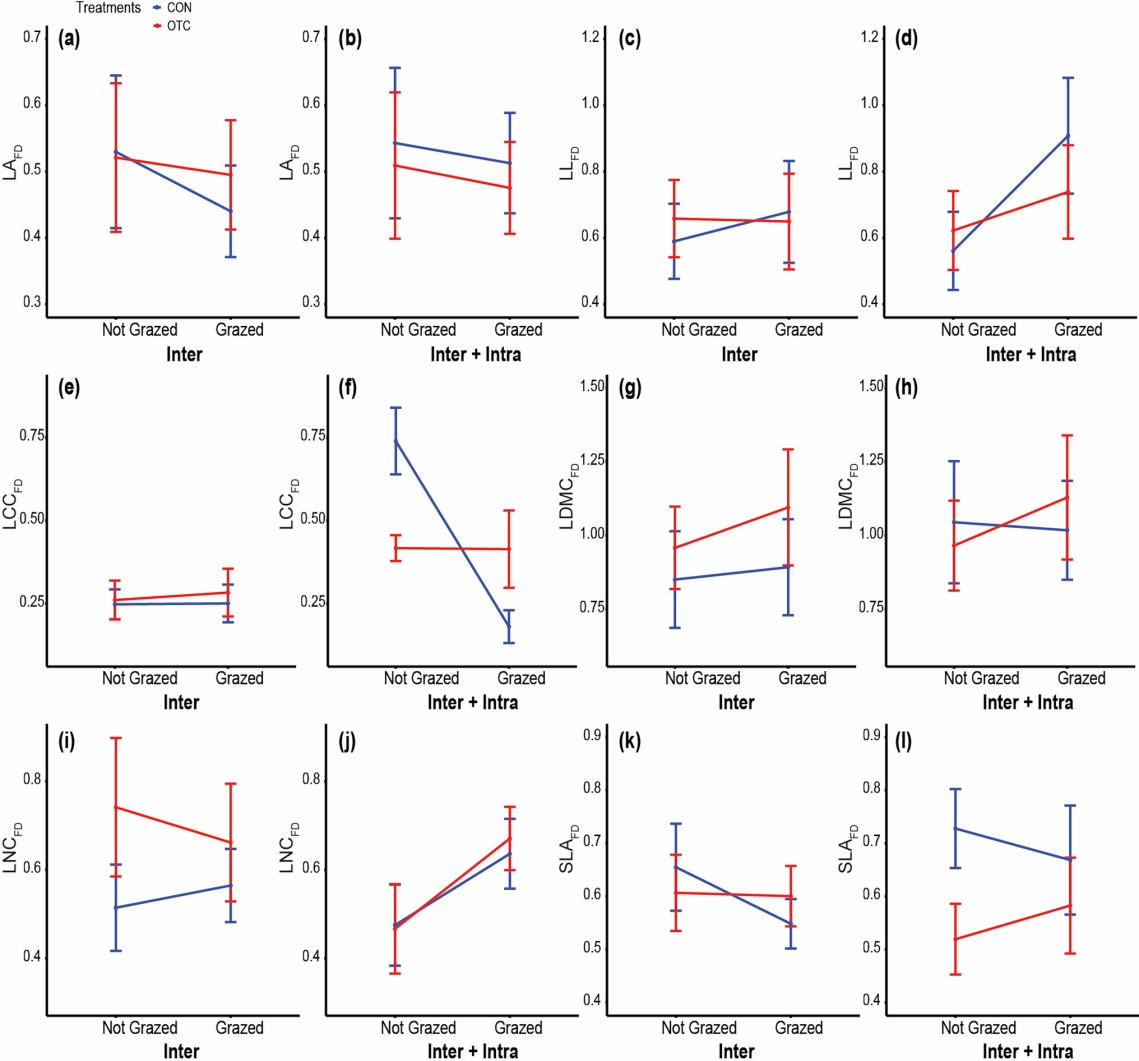
Functional diversity for six leaf functional traits: **a** and **b** Leaf area, **c** and **d** Leaf length, **e** and **f** Leaf carbon content, **g** and **h** Leaf dry matter content, **i** and **j** Leaf nitrogen content, **k** and **l** Specific leaf area, in the four combinations of OTC and grazing. As in Fig. 1, interspecific variability (Inter) averages are based on variation in species abundances but constant trait values per species across treatments, while the interspecific and intraspecific variability averages (Inter + Intra) FD reflect both variation in species abundances and also trait plasticity within a species for a given combination of OTC and grazing. Error bars denote standard errors

Treatment and interaction effects on FD were not strengthened when intraspecific and interspecific variation were combined (Appendix S5). Consequently, no significant main effect of the treatments on FD was detected on four of our six traits when combining both sources of variation (Table 2). A notable exception was observed for LCC and LNC. With the two sources of variation combined, grazing affected LCC_FD_ and LNC_FD_, and there was also an OTC × grazing interaction for LCC_FD_, where OTC decreased LCC_FD_ without grazing and increased it with grazing (Fig. 2).

## Discussion

Our experiment in mountain steppe demonstrates that two attributes of the functional plant-community structure, CWM and FD, show stronger initial responses to warming/aridity during the growing season than to dormant season grazing. We also clearly emphasize the importance of studying these two factors together, although temporally disjunct, because the effect of OTC was primarily observed in the absence of dormant season grazing. Importantly, over the time scale of this experiment, some responses of CWM and FD to the two drivers of global change, and especially to their interactions, were revealed only after accounting for intraspecific trait variation, likely reflecting leaf plasticity. Overall, our results corroborate that trait plasticity is a crucial part of community level functional dynamics. Because responses through interspecific and intraspecific variation were positively correlated, intraspecific trait variation could provide an early indication of future ecosystem responses to environmental changes, especially when multiple drivers are involved.

Harsher environmental conditions are likely to occur in this region in the future, including increased frequency and magnitude of drought combined with warming events (Trenberth et al. 2014; Sarhadi et al. 2018). Such changes in mountain steppes may shift community functional structure through both intraspecific trait variation (Liancourt et al. 2015) and species turnover (Ronk et al. 2020, see also Jung et al. 2014; Siefert et al. 2015; Wang et al. 2022). Notably, the shift in the CWM in response to OTC was observed after only two growing seasons, even without accounting for intraspecific contributions. Although OTC had no main effect on FD for any traits, changes in CWM provided evidence, consistent with our hypothesis, that increased aridity reduces the abundance of species that have larger leaves (reduced LL_CWM_ and LA_CWM_) and traits related to fast carbon and nutrient economy (reduced SLA_CWM_ and LNC_CWM_, increased LDMC_CWM_), the characteristics considered to represent more competitive and less stress tolerant strategies (Pierce et al. 2013). Our results are not fully consistent with the effect of warming alone documented in more mesic conditions, where OTC has been shown to promote plant growth and community greening (i.e., favoring more competitive species, Elmendorf et al. 2012). However, our results are very similar to those from a short-term drought experiment conducted in the French Alps at similar elevation (Jung et al. 2014). We surmise that results could be explained by the fact that our OTC treatment compounds increased temperature and drought. This striking similarity to French Alps study may suggest that the effect of warming with OTC likely magnified the effect of drought and increased the overall level of stress in our system (Liancourt et al. 2012a; Ronk et al. 2020).

The positive, OTC-induced covariation between species turnover and intraspecific trait variation found for CWM, for most traits, is consistent with the general trend observed in a previous global scale analysis (Siefert et al. 2015). Although the effect of OTC on CWM could be detected by accounting for species turnover only, the positive covariation indicates that these two terms reinforce each other. Omitting intraspecific trait variation, as many studies do, would lead to underestimating the magnitude, and especially the pace, of how climate change affects functional plant-community structure.

Just like warming, livestock grazing can affect functional community structure through both species turnover and/or changing intraspecific trait variation (Hedwall et al. 2018). In our case, after grazing exclusion for 1 year, we only detected the latter. In other systems, grazing often increases the representation of less palatable and short-statured species (Dıaz and Cabido 2001; Cingolani et al. 2005; Peco et al. 2012; Diaz et al. 2007). However, because grazing occurred during the dormant season in our experiment, the plasticity we observed likely reflects a response to its influence on abiotic factors. We believe grazing exclusion induced plasticity through indirect effects of litter accumulation and decreased light levels (Facelli and Pickett 1991; Kahmen and Poschlod 2008; Peco et al. 2012; Spence et al. 2014). The main effect of grazing on LL_CWM_ and LNC_FD_, two traits known to respond to shade, align with this interpretation (Evans 1989; Lambers et al. 2008).

There was considerable interaction between grazing and warming as trait responses mainly occurred in the absence of grazing, illustrating the importance of considering climate change in the context of land use (Pyke and Marty 2005; Klein et al 2007; Post and Pedersen 2008; Maestre et al. 2022). Local cessation of grazing is increasingly likely to occur based on contemporary changes in Mongolian lifestyles (Morris and Bruun 2005). The literature suggests similar trait responses to warming as to grazing (Milchunas et al. 1988; Adler et al. 2004; Quiroga et al. 2010; Koerner and Collins 2014), which could compound trait responses when warming and grazing occur together. Our study shows that trait responses to these two factors are non-additive; the OTC effect on the CWM was much stronger in the absence of grazing, well illustrating the difficulty of predicting combined effects of two drivers (Klein et al. 2007; Kohli et al. 2021). It is also the case that the interactive effect of increased aridity and dormant season grazing on CWM becomes apparent only when both inter- and intraspecific variation are considered together. Thus, at least initially, one driver may act on functional community structure through changes in species turnover (e.g., warming/aridity), but its consequences can be mitigated by another driver acting through intraspecific variation (e.g., grazing).

Our results highlight two key aspects of the effects of global changes on plant communities. First, we showed how the expression of functional traits in response to one driver can be modified by another, even when the two are temporally disjunct. In Mongolian steppe, climate change may have a more pronounced effect on the functional community structure if dormant season grazing ceases. Second, while treatment-induced changes in community level functional traits were explained by a combination of both interspecific and intraspecific trait variation, the latter contributed considerably to CWM and FD. Trait plasticity (or the lack of it) precedes and possibly predicts changes in species relative abundance (Liancourt et al. 2015), which, in turn, precedes species turnover (Suding et al. 2008). Viewed in the context of climate change, intraspecific trait variation may provide early signals of long-term community changes or resilience. As many plant species are unlikely to disperse fast enough to track the rapidly changing climate, trait plasticity may figure importantly in community responses to global changes (Nicotra et al. 2010).

### Supplementary Information

Below is the link to the electronic supplementary material.

Supplementary file1 (DOCX 147 KB)

## Declarations

### Availability of data and material

The datasets used and/or analyzed during the current study are available from the corresponding author on reasonable request.
