# Supplementary material for: Leaf trait plasticity reveals interactive effects of temporally disjunct grazing and warming on plant communities
Source: Oecologia. 2024 Apr 4;204(4):833–43. doi: 10.1007/s00442-024-05540-z (PMC11062997; doi:10.1007/s00442-024-05540-z)
Supplement: Supplementary file 1 — Supplementary file1 (DOCX 147 KB) [file 442_2024_5540_MOESM1_ESM.docx]

**Intraspecific trait variation reveals interaction between winter grazing and summer warming**

**Appendix S1. Principal Components Analysis (PCA) of leaf functional traits**


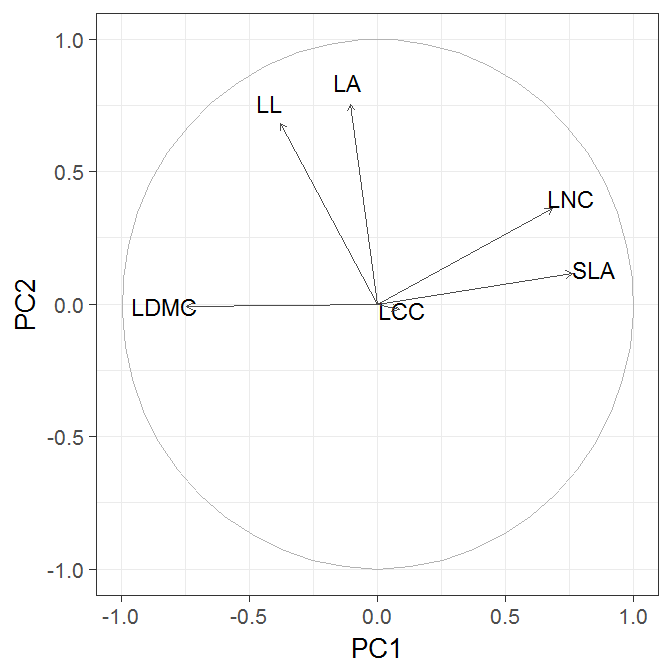


**Fig S1.** Variables factor map of leaf functional traits: leaf area per leaf (LA), specific leaf area (SLA), leaf length (LL), leaf dry matter content (LDMC), leaf nitrogen content per unit leaf mass (LNC), and leaf carbon content per unit leaf mass (LCC) in control plots.

**Appendix S2. Species analyzed for leaf functional traits.**

**Table S1**. Species found within the experiment from 2009-2010 and analyzed for six leaf functional traits.

| **Species** | **Graminoid/Forb** | **Family** |
| --- | --- | --- |
| *Agropyron cristatum* | Graminoid | Poaceae |
| *Agrostis mongholica* | Graminoid | Poaceae |
| *Amblynotus rupestris* | Forb | Boraginaceae |
| *Arenaria capillaris* | Forb | Caryophyllaceae |
| *Artemisia commutata* | Forb | Asteraceae |
| *Artemisia tanacetifolia* | Forb | Asteraceae |
| *Aster alpinus* | Forb | Asteraceae |
| *Astragalus inopinatus* | Forb | Fabaceae |
| *Astragalus oroboides* | Forb | Fabaceae |
| *Astragalus* spp. | Forb | Fabaceae |
| *Bupleurum bicaule* | Forb | Apiaceae |
| *Carex* spp. | Graminoid | Cyperaceae |
| *Chrysanthemum zawadskii* | Forb | Asteraceae |
| *Dianthus versicolor* | Forb | Caryophyllaceae |
| *Elymus chinensis* | Graminoid | Poaceae |
| *Festuca lenensis* | Graminoid | Poaceae |
| *Galium verum* | Forb | Rubiaceae |
| *Helictotrichon schellianum* | Graminoid | Poaceae |
| *Hierochloe odorata* | Graminoid | Poaceae |
| *Koeleria macrantha* | Graminoid | Poaceae |
| *Leontopodium leontopodioides* | Forb | Asteraceae |
| *Oxytropis strobilacea* | Forb | Fabaceae |
| *Oxytropis viridiflava* | Forb | Fabaceae |
| *Polygonum angustifolium* | Forb | Polygonaceae |
| *Potentila sericea* | Forb | Rosaceae |
| *Potentilla acaulis* | Forb | Rosaceae |
| *Potentilla bifurca* | Forb | Rosaceae |
| *Potentilla fragarioides* | Forb | Rosaceae |
| *Potentilla tanacetifolia* | Forb | Rosaceae |
| *Pulsatilla turczaninovii* | Forb | Ranunculaceae |
| *Sanguisorba officinalis* | Forb | Rosaceae |
| *Scabiosa comosa* | Forb | Dipsacaceae |
| *Sibbaldianthe adpressa* | Forb | Rosaceae |
| *Thalictrum minus* | Forb | Ranunculaceae |
| *Thymus gobicus* | Forb | Lamiaceae |
| *Veronica incana* | Forb | Plantaginaceae |

**Appendix S3. Explained variance by individual factors for CWM**

**Table S2.** Results of two-way ANOVAs for CWM in six leaf functional traits. Explained variance by individual factors in the experiment for individual components of plant communities’ functional structure (%).

|  |  |  | Inter |  | Intra |  | Covariation |  | Inter + Intra |
| --- | --- | --- | --- | --- | --- | --- | --- | --- | --- |
| ***Leaf size*** | OTC |  | 5.98 |  | 2.08 |  | 7.05 |  | 15.10 |
|  | Grazing |  | 2.19 |  | 1.34 |  | 3.42 |  | 6.95 |
| **LA CWM** | OTC x Grazing |  | 1.02 |  | 8.95 |  | 6.06 |  | 16.03 |
|  | Residuals |  | 37.47 |  | 18.09 |  | 6.37 |  | 61.92 |
|  | Total |  | 46.66 |  | 30.45 |  | 22.89 |  | 100 |
|  |  |  |  |  |  |  |  |  |  |
|  | OTC |  | 4.10 |  | 4.27 |  | 8.38 |  | 16.75 |
|  | Grazing |  | 2.35 |  | 2.22 |  | 4.57 |  | 9.13 |
| **LL CWM** | OTC x Grazing |  | 1.21 |  | 9.43 |  | 6.75 |  | 17.38 |
|  | Residuals |  | 38.60 |  | 13.12 |  | 5.02 |  | 56.73 |
|  | Total |  | 46.26 |  | 29.03 |  | 24.71 |  | 100 |
| ***Carbon***  ***and nitrogen economy*** |  |  |  |  |  |  |  |  |  |
|  | OTC |  | 6.65 |  | 3.50 |  | 9.65 |  | 19.81 |
|  | Grazing |  | 3.97 |  | 0.01 |  | -0.43 |  | 3.55 |
| **LCC CWM** | OTC x Grazing |  | 2.17 |  | 9.85 |  | 9.24 |  | 21.25 |
|  | Residuals |  | 34.51 |  | 16.10 |  | 4.78 |  | 55.40 |
|  | Total |  | 47.30 |  | 29.47 |  | 23.24 |  | 100 |
|  |  |  |  |  |  |  |  |  |  |
|  | OTC |  | 6.68 |  | 3.53 |  | 9.71 |  | 19.91 |
|  | Grazing |  | 2.45 |  | 0.09 |  | -0.94 |  | 1.60 |
| **LDMC CWM** | OTC x Grazing |  | 1.49 |  | 13.61 |  | 9.01 |  | 24.12 |
|  | Residuals |  | 33.40 |  | 25.25 |  | -4.28 |  | 54.37 |
|  | Total |  | 44.02 |  | 42.48 |  | 13.50 |  | 100 |
|  |  |  |  |  |  |  |  |  |  |
|  | OTC |  | 3.38 |  | 12.16 |  | 12.82 |  | 28.36 |
|  | Grazing |  | 2.13 |  | 0.83 |  | -2.66 |  | 0.30 |
| **LNC CWM** | OTC x Grazing |  | 1.59 |  | 11.99 |  | 8.73 |  | 22.31 |
|  | Residuals |  | 20.54 |  | 21.57 |  | 6.91 |  | 49.02 |
|  | Total |  | 27.64 |  | 46.55 |  | 25.81 |  | 100 |
|  |  |  |  |  |  |  |  |  |  |
|  | OTC |  | 6.0 |  | 5.05 |  | 11.01 |  | 22.05 |
|  | Grazing |  | 3.64 |  | <0.001 |  | -0.05 |  | 3.60 |
| **SLA CWM** | OTC x Grazing |  | 2.60 |  | 13.52 |  | 11.87 |  | 27.99 |
|  | Residuals |  | 29.47 |  | 15.51 |  | 1.38 |  | 46.36 |
|  | Total |  | 41.72 |  | 34.07 |  | 24.21 |  | 100 |

**Appendix S4. Variation Intra for FD**

**Fig. S2.** Functional diversity expressed through intraspecific variation only for six leaf functional traits showing the significant OTC × grazing interaction for LA_FD_, LL_FD_ and LCC_FD_, the significant main effect of OTC on LDMC_FD_ and SLA_FD_, and the main effect of grazing cessation on LNC_FD_.

**Appendix S5. Explained variance by individual factors for FD**

|  |  |  | Inter |  | Intra |  | Covariation |  | Inter + Intra |
| --- | --- | --- | --- | --- | --- | --- | --- | --- | --- |
| ***Leaf size*** |  |  |  |  |  |  |  |  |  |
|  | OTC |  | 0.21 |  | 1.37 |  | -1.08 |  | 0.50 |
|  | Grazing |  | 1.33 |  | 0.27 |  | -1.19 |  | 0.40 |
| **LA FD** | OTC x Grazing |  | 0.40 |  | 0.45 |  | -0.85 |  | 0.001 |
|  | Residuals |  | 104.0 |  | 1.90 |  | -6.82 |  | 99.09 |
|  | Total |  | 105.9 |  | 3.98 |  | -9.95 |  | 100 |
|  |  |  |  |  |  |  |  |  |  |
|  | OTC |  | 0.06 |  | 0.88 |  | -0.47 |  | 0.47 |
|  | Grazing |  | 0.26 |  | 5.94 |  | 2.48 |  | 8.69 |
| **LL FD** | OTC x Grazing |  | 0.39 |  | 0.71 |  | 1.05 |  | 2.15 |
|  | Residuals |  | 80.11 |  | 1.88 |  | 6.70 |  | 88.69 |
|  | Total |  | 80.82 |  | 9.42 |  | 9.76 |  | 100 |
| ***Carbon***  ***and nitrogen economy*** |  |  |  |  |  |  |  |  |  |
|  | OTC |  | 0.15 |  | 1.29 |  | -0.87 |  | 0.56 |
|  | Grazing |  | 0.05 |  | 24.54 |  | -2.10 |  | 22.48 |
| **LCC FD** | OTC x Grazing |  | 0.03 |  | 20.45 |  | 1.52 |  | 22.00 |
|  | Residuals |  | 27.60 |  | 16.72 |  | 10.64 |  | 54.95 |
|  | Total |  | 27.82 |  | 62.99 |  | 9.19 |  | 100 |
|  |  |  |  |  |  |  |  |  |  |
|  | OTC |  | 0.03 |  | 2.37 |  | 0.55 |  | 2.95 |
|  | Grazing |  | 0.57 |  | 0.06 |  | 0.36 |  | 0.98 |
| **LDMC FD** | OTC x Grazing |  | 1.11 |  | 0.28 |  | -1.11 |  | 0.28 |
|  | Residuals |  | 119.1 |  | 3.79 |  | -27.08 |  | 95.79 |
|  | Total |  | 120.8 |  | 6.5 |  | -27.29 |  | 100 |
|  |  |  |  |  |  |  |  |  |  |
|  | OTC |  | 10.78 |  | 9.11 |  | -19.83 |  | 0.07 |
|  | Grazing |  | 0.09 |  | 16.03 |  | -2.39 |  | 13.73 |
| **LNC FD** | OTC x Grazing |  | 1.73 |  | 3.07 |  | -4.62 |  | 0.19 |
|  | Residuals |  | 168.3 |  | 45.37 |  | -127.6 |  | 86.01 |
|  | Total |  | 180.9 |  | 73.59 |  | -154.5 |  | 100 |
|  |  |  |  |  |  |  |  |  |  |
|  | OTC |  | 0.001 |  | 9.81 |  | -0.24 |  | 9.57 |
|  | Grazing |  | 1.41 |  | 1.51 |  | -2.92 |  | 0.002 |
| **SLA FD** | OTC x Grazing |  | 1.11 |  | 0.06 |  | 0.50 |  | 1.67 |
|  | Residuals |  | 53.53 |  | 31.41 |  | 3.82 |  | 88.76 |
|  | Total |  | 56.05 |  | 42.79 |  | 1.16 |  | 100 |

**Table S3.** Results of two-way ANOVAs for FD in six leaf functional traits. Explained variance by individual factors in the experiment for individual components of plant communities’ functional structure (%).
